# Development and Implementation of Couple-Based Collaborative Management Model of Type 2 Diabetes Mellitus for Community-Dwelling Chinese Older Adults: A Pilot Randomized Trial

**DOI:** 10.3389/fpubh.2021.686282

**Published:** 2021-07-13

**Authors:** Yuyang Liu, Xiaocun Xiao, Chaonan Peng, Tianyi Zhao, Yanjuan Wu, Wanwen Yu, Liping Ou, Xiongfei Chen, Xueji Wu, Dong Roman Xu, Jing Liao

**Affiliations:** ^1^Department of Medical Statistics, School of Public Health, Sun Yat-sen University, Guangzhou, China; ^2^Sun Yat-sen Global Health Institute, School of Public Health and Institute of State Governance, Sun Yat-sen University, Guangzhou, China; ^3^School of Nursing, Sun Yat-sen University, Guangzhou, China; ^4^Qichuang Social Work Service, Guangzhou, China; ^5^Panyu District Community Health Service Management Centre, Guangzhou, China; ^6^Division of Primary Health Care, Guangzhou Centre for Disease Control and Prevention, Guangzhou, China; ^7^School of Health Management, Southern Medical University, Guangzhou, China

**Keywords:** coupled-based intervention, chronic disease management, model development, feasibility, implementation assessment

## Abstract

**Background:** To mobilize family's positive involvement in improving and sustaining self-management activities of older adults with diabetes, we developed a couple-based collaborative management model (CCMM) for community-dwelling older Chinese.

**Methods:** The model was developed stepwise through applying theoretical models, interviewing older couples and community healthcare workers, as well as incorporating expert reviews. A 3-month pilot study was conducted to test the model's feasibility and its treatment effects by linear regression on 18 pairs of older couples aged 60 years+, who were equally divided into a couple-based intervention arm and a patient-only control arm.

**Results:** The developed CCMM covered four theory-driven intervention modules: dyadic assessment, dyadic education, dyadic behavior-change training, and dyadic monitoring. Each module was delivered by community healthcare workers and targeted at older couples as the management units. Based on interviews with older couples and healthcare workers, 4 weekly education and training group sessions and 2-month weekly behavior change booster calls were designed to address older adults' main management barriers. These modules and session contents were evaluated as essential and relevant by the expert panel. Furthermore, the CCMM showed good feasibility and acceptability in the pilot, with non-significant yet more positive changes in physiological outcomes of diabetic participants and couples' well-being and exercise levels of these in the intervention arm than their controlled counterparts.

**Conclusion:** We systematically developed a couple-based collaborative management model of diabetes, which was well-received by healthcare practitioners and highly feasible among older Chinese couples living in the community. The model's treatment effects need to be verified in fully powered randomized controlled trials.

**Clinical Trial Registration:** http://www.chictr.org.cn/showproj.aspx?proj=42964, identifier: ChiCTR1900027137.

## Introduction

China's ever-increasing diabetes burden of the older population exceeds its capacity of healthcare services (1). Under the coronavirus disease 2019 (COVID-19) pandemic, prioritizing healthcare resources and personnel into infectious disease control and treatment as well as national level quarantine measures made it even difficult for older adults with diabetes to maintain their routine treatment and healthcare services (2). Although self-management has long been recommended by Chinese clinical guidelines as an effective manner to control blood glucose levels and prevent complications, adherence to self-management activities is generally low among older adults in China (3, 4). Diabetes self-management requires lifelong commitments to multiple care regimens, which permeate daily routines and interacts with living context (5). Evidence-based interventions addressing behavioral and environmental barriers should be identified to promote self-management activities of older adults with diabetes.

Accumulating evidence has suggested that family-engaged interventions, especially these motivating support from the spouse (6), significantly improved the self-management activities of older people with diabetes (7). Marital relationship is a well-established factor for couples' health that should be considered and leveraged in disease management (7). Previous couple-based interventions on chronic disease management have revealed significant treatment effects on the depressive symptom, pain, and marital functioning of the patients (8), while evidence has been inconclusive regarding improvements in the patients' or their spouse's physical health (9–11) or their health behavior (12).

Less is clear about the effects of couple-based interventions on diabetes management. Our scoping review on couple-based random controlled trials (RCTs) of type 2 diabetes mellitus (T2DM)^1^ showed insufficient evidence neither on glucose control (13, 14) or changes in self-management activities of the participants with diabetes (14, 15), nor significant changes in diet or exercise levels of their spouse (16). Most of these studies provided no theoretical bases for their intervention design and were subjected to implementation and reporting bias. Further studies equipped with theory-driven interventions and outcome measures targeting at both the participants with diabetes and their spouse are needed to clarify the treatment effects of couple-based diabetes management (10).

This paper was an interim report of the project on the couple-based collaborative management model (CCMM) of T2DM for community-dwelling older adults in China, which is a community-based multicenter RCT aiming to systematically develop and validate a CCMM integrating health professionals and family supporters (17). This report provided detailed information on the development of CCMM, grounded in theories, and tailored to the care needs of older Chinese couples with T2DM.

## Materials and Methods

### Step 1: Theory-Driven Model Development

The CCMM was developed based on Berg and Upchurch's dyadic model of coping with chronic illness (DMCCI) (18) and Bandura's social cognitive theory (SCT) (19). DMCCI describes couples' dyadic appraisal of the illness severity, ownership and management responsibility, and dyadic coping strategies ranging from uninvolved (patient coping alone), supportive (spouse taking a supportive role), collaborative (spouse actively involved and the couple coping jointly) to control (spouse dominating the care responsibility) (18). If the couple both appraised the chronic disease as a shared problem needed to be coped together, a communal coping would be formed (20), which would boost their collective efficacy in managing chronic diseases, in addition to the patient's self-efficacy emphasized in the SCT (19). The collective efficacy of the couple can be strengthened by successful experiences of jointly developing and achieving management goals, alternative experiences such as acting as each other's role model, as well as language persuasion and positive physiological feedbacks (21). Intervention modules targeted at these key theoretical components were formed, with intervention contents further developed in line with the community diabetes self-management education program (22) and the Chinese clinical guideline for T2DM prevention and treatment (3). The community healthcare centers' current services were also integrated into CCMM to maximize its normalization into their routine practices.

### Step 2: Management Barriers Assessment of Older Adults With Diabetes

To ensure intervention contents of CCMM that met the management needs of older couples, we conducted semistructured group interviews among couples aged 60+ years with at least one partner having diabetes and community health workers involving in diabetes care. Interviews were conducted between March to August 2019 in Guangzhou, Guangdong, China. These interviews explored couples' main barriers in daily diabetes management from couples' and care managers' perspectives. Each one and half an hour interview was composed of three researchers (a host, a recorder, and an observer) and five to six couples or seven to eight community health workers. Interviews were audio-recorded and transcribed verbatim. Detailed qualitative analyses of these interviews are presented in separate papers of the series of studies (5). The study was approved by the Institutional Review Board of the School of Public Health of Sun Yat-sen University (#SYSU 2019-064), and the drafting of this manuscript adheres to the CONSORT statement (Supplementary Table 1) (23). All participants read and signed the written informed consent approved by the institutional review board prior to participation.

### Step 3: Incorporation of Expert Reviews

To further tailor CCMM to the focus of chronic disease management in China, the expert reviews on the intervention content of model were conducted following a Delphi approach. An independent panel was convened, including 11 experts that consist of diabetes clinicians, general practitioners, community chronic disease management officials, and social workers. Every expert assessed the content of CCMM for its necessity, relevance, and clarity with quality reviews and quantity measures (24). The evaluation was synthesized and revaluated until the panel reached 80% consensus.

### Step 4: Feasibility Study

A 3-month feasibility study among older couples was carried out between September and December 2019. Participants with diabetes were recruited from a community of Guangzhou by community healthcare workers if they had T2DM, with the latest fasting blood glucose level > 8.0 mmol/L or glycosylated hemoglobin (HbA1c) > 7.0%, aged 60+ years, cohabited with spouses, and normal cognitive and behavioral capacity. Participants with diabetes who previously participated in a similar education group were excluded. Their cohabited spouses with no mental or physical dysfunction were also recruited. In the pilot study, the partner with higher baseline blood glucose was treated as “participants with diabetes” if the couple pair both had diabetes. All participants provided informed consent.

Out of the 24 pairs of couple screened with signed informed consent, 18 eligible couple pairs were enrolled (recruitment rate = 75%) and 1:1 simple randomized into couple-based intervention arm (*n* = 9) and individual-based control arm (*n* = 9) by an uninformed researcher. The content of interventions was delivered by two community healthcare workers targeted at both the diabetic participants and their spouses for the intervention arm while only at the diabetic participants for the control arm (17). After the interventions, semistructured group interviews were conducted to evaluate the intervention's feasibility by the couples of the intervention arm and community healthcare workers. Two-month weekly behavior change booster calls were implemented afterwards.

### Study Measures

To examine CCMM's treatment effects, we measured couples' physiological health [i.e., HbA1c as primary outcome measures for patients, fasting blood glucose, lipid profiles, and body mass index (BMI)], psychological health (i.e., quality of life by the 36-item short form survey as primary outcome measures for spouses, self-efficacy by the Chinese version of the diabetes management questionnaire), and self-management behaviors by the summary of diabetes self-care activities questionnaire and physical activity by the international physical activity questionnaire-short form at baseline and 3 months after the beginning of the intervention. Detailed outcome measures were provided in the study protocol (17).

The CCMM's feasibility was evaluated quantitatively by the attendance rates of education sessions, follow-up rates of booster calls, and acceptability scores of couples of intervention and participants with diabetes of control arm and qualitatively by semistructured group interviews with couples of the intervention arm and community health workers who delivered CCMM, guided by the normalization process theory (NPT) (interview script in Supplementary Table 2) (25).

### Statistical Analyses

Intention-to-treat was applied to examine the treatment effects for the diabetic participants and spouses separately. Six percent to 6–17% couples missed the psychological measures, and 11–22% participants with diabetes missed the physiological measures (detailed information see Supplementary Table 3). Missing values were multiply imputed under the missing at the random assumption by intervention and control arms separately by arms to avoid biasing treatment effects toward the null (26). Fifty complete datasets for each arm were multiply imputed using individual demographic information, all physiological measures, and all psychological measures. Within-arm changes in health measures between baseline and 3 months were calculated by paired *t*-tests. Between-arm differences in the changes in health outcomes were compared by linear regression. Furthermore, differences in diabetic participants across arms and correlations between couples of the intervention arm in the implementation measures and acceptability scores were examined by Wilcoxon rank-sum test and Spearman's rank correlation, respectively, because of small sample size and non-normal distribution. All data analyses were by R Version 3.61.

## Results

### Step 1: Theory-Driven Model Development

**Figure 1:**
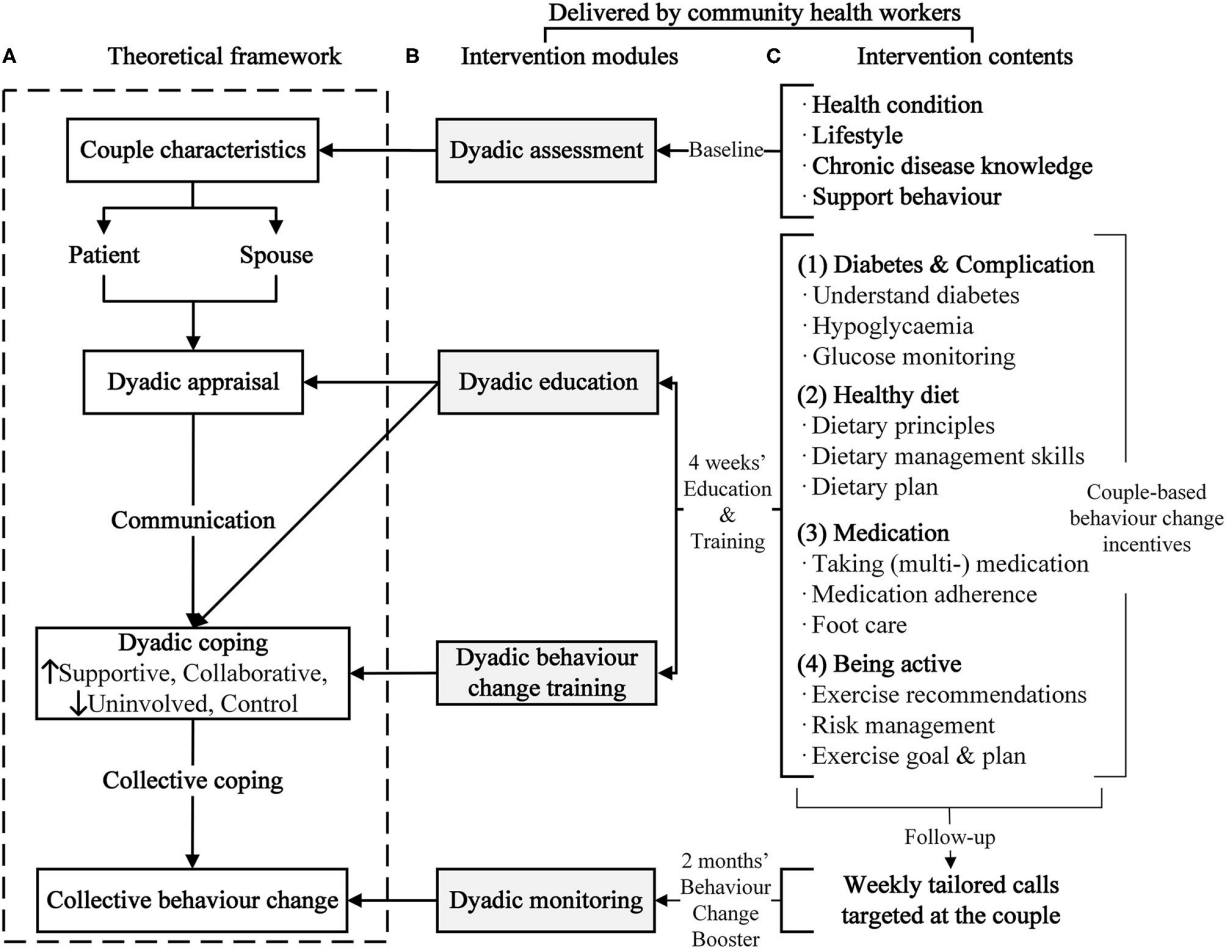
Theoretical framework and modules of couple-based collaborative management model. **(A)** Theoretical framework. **(B)** Intervention modules. **(C)** Intervention contents.

As shown in the CCMM theoretical framework (Figures 1A,B), the intervention prototype covered four modules: (1) dyadic assessment to evaluate older couples' baseline sociodemographic characteristics, health status, and lifestyle as the evidence base of intervention context; (2) dyadic education to address older couple's dyadic appraisal and understanding about diabetes management *via* group education base on the background of dyadic assessment; (3) dyadic behavior change training to improve couples' dyadic coping skills by promoting collaborative action for behavior change by using experience, encouragement, and positive feedback, which can enhance not only the efficacy of older adults with diabetes but also the collective behavior of couples; (4) dyadic monitoring to assess and provide timely feedback to improve dyadic behavior health. All modules had community health workers serving as care managers and older couples as management units.

### Step 2: Management Barriers Assessment of Older Adults With Diabetes

Altogether, we interviewed 11 pairs of older couples with T2DM and 33 community health workers from four community healthcare centers. These interviews helped identify barriers in daily T2DM management from the perspectives of older adults with diabetes, spouses, and clinicians. The main barriers of older couples with diabetes included lack of knowledge about diabetes and complications prevention, short of authentic information on medication, and lack of support to properly conduct self-management activities, particularly regarding foot care, healthy diet, and exercise. Couples trusted community health workers who understand their condition at most and assessed them as the best suitable group delivered personalized education mentioned above. To make use of this information, we invited health workers as care managers for four modules. To address these management barriers, the intervention contents of education and training sessions were designed to improve the knowledge and skills of (1) diabetes and complications management, (2) healthy diet, (3) medication adherence, and (4) exercise (Figure 1C). To facilitate older couples to adopt healthy behaviors, each session further employed behavior change techniques (weekly and special training) and couple-based behavior change incentives (material and social incentives and rewards based on couples' performances). A detailed information of change techniques and incentives was shown in our protocol.

### Step 3: Incorporation of Expert Reviews

The expert panel well-received the context of CCMM, evaluated as essential (the average of content validity ratio was 0.89, with a minimum acceptable value of 0.59) and relevant (the item-level content validity index was 0.72–1, and the average and union agreement of scale-level content validity index was 0.96 and 0.77, respectively, with a generally accepted value of 0.80). They commented that most of the intervention contents were well-organized and clearly presented, except for some sections that need to be tailored to the actual situation of people with diabetes (e.g., knowledge of insulin may only be useful for those using insulin). The panel further suggested that the course contents should be easily understandable by less-educated older couples. Considering expert reviews, we simplified educational content by deleting low essential and replaced one-way education with interactive activities for older couples to practice together.

### Step 4: Feasibility Study

Older couples' sociodemographic characteristics were comparable between arms (Table 1). Participants with diabetes had a mean age of 70 years, half were male, had low education, and the majority were retired. They had diabetes for multiple years, and over two-thirds had diseases other than diabetes. Spouses had similar sociodemographic profiles, who tended to be younger and better educated.

**Table 1:** Couples' sociodemographic characteristics of the 3-month pilot study (couple pairs *N* = 18).

**Variables**	**Participants with diabetes**		**Spouses**	
	**Intervention arm** **(*n* = 9)**	**Control arm** **(*n* = 9)**	**Intervention arm** **(*n* = 9)**	**Control arm** ** (*n* = 9)**
**Sociodemographic characteristics**				
Age (years), mean (SD)	73.2 (8.8)	70.3 (5.5)	71.3 (8.8)	68.9 (7.1)
Male, *n* (%)	5 (56)	4 (44)	4 (44)	5 (56)
Primary school and below, *n* (%)	6 (67)	5 (56)	3 (33)	3 (33)
Retired, *n* (%)	8 (89)	9 (100)	9 (100)	9 (100)
Diabetes duration (years), mean (SD)	11.1 (4.5)	8.8 (7.8)	N/A	N/A
Had other diagnosed diseases,^a^ *n* (%)	7 (78)	6 (67)	4 (44)	8 (89)

*SD, standard deviation*.

a *Diagnosed disease included hypertension, hyperlipidemia, cardiopathy, coronary artery disease, heart failure, kidney diseases, cerebrovascular diseases, and gout*.

The models' treatment effects were shown in Table 2 for the diabetic participants and Table 3 for the spouses. Although none of these treatment effects were statistically significant due to insufficient power (e.g., power for HbA1c was 0.1), trends in within- and between-arms differences of these measures were large as expected; namely, participants with diabetes of the intervention arm tended to have faster declines in blood glucose and lipid levels, more improvements in mental well-being, self-efficacy, and physical activities than their controlled counterparts. Nevertheless, participants with diabetes of the control arm seemed to have better outcomes in terms of BMI, self-reported physical health, and daily management activities. Similar trends showed among the spouses. Notably, spouses of the intervention arm reported increased confidence in assisting their partners in diabetes management, while an opposite tendency was shown among spouses of the control arm.

**Table 2:** Treatment effects of couple-based interventions on participants with diabetes of the 3-month pilot study (couple pair *N* = 18).

**Variables of participants with diabetes**	**Intervention arm (*****n*** **=** **9)**				**Control arm (*****n*** **=** **9)**				**Treatment effect**	
	**Baseline^**a**^**	**3-month^**a**^**	**Difference within arm^**b**^**	***P^***c***^***	**Baseline^**a**^**	**3-month^**a**^**	**Difference within arm^**b**^**	***P^***c***^***	**Difference between arm^**d**^**	***P^***c***^***
	**Mean (SD)**	**Mean (SD)**	**Mean (SE)**		**Mean (SD)**	**Mean (SD)**	**Mean (SE)**		**Mean (SE)**	
**Physiological outcomes**										
HbA_1c_ (%)	8.6 (2.1)	8.1 (2.0)	−0.3 (0.5)	0.586	7.9 (1.5)	7.8 (1.6)	−0.2 (0.4)	0.656	−0.1 (0.6)	0.848
FBG (mmol/L)	9.5 (2.3)	9.2 (2.4)	−0.3 (1.0)	0.776	8.4 (2.2)	8.6 (2.5)	0.3 (0.9)	0.781	−0.6 (1.4)	0.686
BMI (kg/m^2^)	24.0 (1.3)	25.0 (2.1)	1.0 (0.7)	0.201	24.8 (2.8)	24.9 (3.1)	0.1 (0.5)	0.822	1.0 (0.9)	0.286
Total cholesterol (mmol/L)	9.8 (15.1)	5.0 (0.8)	−4.8 (4.9)	0.367	4.4 (1.0)	4.1 (0.8)	−0.2 (0.3)	0.568	−4.6 (5.0)	0.370
Triglycerides (mmol/L)	2.1 (1.0)	2.3 (1.3)	0.2 (0.4)	0.658	1.8 (1.5)	1.8 (1.4)	−0.0 (4.7)	0.987	0.2 (0.6)	0.763
LDL-C (mmol/L)	2.8 (1.0)	1.2 (0.2)	−1.7 (0.3)	0.001	2.5 (0.9)	1.3 (0.2)	−1.2 (0.3)	0.005	−0.5 (0.4)	0.415
HDL-C (mmol/L)	1.2 (0.2)	2.9 (0.9)	1.7 (0.3)	0.001	1.4 (0.3)	2.0 (0.6)	0.7 (0.3)	0.053	0.9 (0.4)	0.037
**Psychosocial outcomes**										
SF-36: Physical component score	49.9 (10.0)	49.7 (12.3)	0.5 (2.9)	0.868	48.6 (8.3)	49.7 (5.6)	1.0 (2.0)	0.621	−0.5 (3.5)	0.887
SF-36: Mental component score	53.0 (3.7)	53.0 (5.1)	−0.1 (2.5)	0.961	53.7 (3.8)	53.1 (2.8)	−0.6 (0.9)	0.564	0.4 (2.6)	0.872
C-DMQ score	91.6 (14.6)	101.2 (18.0)	9.7 (5.7)	0.135	82.1 (12.5)	91.6 (13.9)	9.4 (4.8)	0.092	0.2 (7.4)	0.976
**Behavior outcomes**										
SADCA score	33.4 (11.1)	38.2 (12.2)	4.8 (3.8)	0.252	32.4 (9.7)	38.2 (10.9)	5.8 (5.3)	0.310	−1.0 (6.5)	0.880
**Metabolic equivalent scores measured by IPAQ-C (MET min week**^**−1**^**)**										
Exercise^e^	594.3 (797.1)	1,440.0 (2,459.3)	676.4 (723.8)	0.384	840.0 (1,313.3)	573.3 (857.8)	−62.6 (628.9)	0.924	739.0 (961.6)	0.456
Walking	1,277.6 (765.1)	1,254.0 (1,372.2)	−167.6 (294.7)	0.560	1,328.3 (635.8)	1,457.5 (893.9)	340.4 (486.9)	0.512	−507.9 (572.8)	0.392
Sitting	900.0 (921.1)	660.0 (761.3)	−51.3 (517.7)	0.924	945.0 (736.5)	1,120.0 (525.7)	378.0 (397.0)	0.381	−429.3 (653.6)	0.523

*SD, standard deviation; SE, standard error; 95% CI, 95% confidence interval; HbA1c, glycosylated; FBG, fasting blood glucose; BMI, body mass index; LDL-C, low-density lipoprotein cholesterol; HDL-C, high-density lipoprotein cholesterol; SF-36, the 36-item Short Form Survey (SF-36); SADCA, the Summary of Diabetes Self-Care Activities questionnaire; IPAQ-C, International Physical Activity Questionnaire—Chinese version; MET min week^−1^, minutes of metabolic equivalent per week for physical activity; C-DMQ, the Chinese version of the Diabetes Management Questionnaires*.

a *The statistical description was based on complete cases without imputation*.

b *The within-arm difference was calculated as the difference between 3-month and baseline levels of given measures using the multiply imputed data*.

c *P-value of the within-arm difference was tested by paired t-test, and the between-arm difference was tested by Student's t-test*.

d *The between-arm difference was calculated as the difference between intervention and control arms using the multiply imputed data*.

e *The exercise was defined as moderate to vigorous physical activity measured by IPAQ-C regarding weekly frequency and duration*.

**Table 3:** Treatment effects of couple-based interventions on spouses of the 3-month pilot study (couple pair *N* = 18).

**Variables of spouses**	**Intervention arm (*****n*** **=** **9)**				**Control arm (*****n*** **=** **9)**				**Treatment effect**	
	**Baseline^**a**^**	**3-month^**a**^**	**Difference within arm^**b**^**	***P^***c***^***	**Baseline^**a**^**	**3-month^**a**^**	**Difference within arm^**b**^**	***P^***c***^***	**Difference between arm^**d**^**	***P^***c***^***
	**Mean (SD)**	**Mean (SD)**	**Mean (SE)**		**Mean (SD)**	**Mean (SD)**	**Mean (SE)**		**Mean (SE)**	
**Psychosocial outcomes**										
SF-36: Physical component score	47.0 (7.7)	46.0 (10.2)	−1.0 (2.9)	0.754	49.1 (9.4)	50.2 (7.4)	0.8 (2.6)	0.761	−1.8 (4.0)	0.657
SF-36: Mental component score	55.0 (4.5)	53.2 (5.3)	−2.0 (2.1)	0.365	56.7 (6.4)	51.8 (5.0)	−5.1 (s2.0)	0.041	3.1 (2.8)	0.297
C-DMQ score	76.7 (30.0)	84.5 (28.5)	10.0 (12.8)	0.467	87.0 (29.2)	75.8 (24.6)	−11.3 (9.8)	0.293	21.3 (16.1)	0.212
**Behavior outcomes**										
Metabolic equivalent scores measured by IPAQ-C (MET min week^−1^)										
Exercise^e^	440.0 (643.7)	1,500.9 (1,530.9)	1,131.2 (607.0)	0.112	1,680.0 (1,680.0)	2,425.7 (3,607.6)	695.0 (869.0)	0.454	436.3 (1,056.1)	0.686
Walking	1,666.5 (874.2)	1,287.0 (1,034.0)	−454.8 (564.3)	0.450	2,277.0 (1,369.4)	1,155.0 (832.9)	−1,043.1 (484.3)	0.073	588.3 (737.7)	0.439
Sitting	1,015.0 (585.2)	805.0 (708.0)	−360.7 (308.6)	0.300	1,560.0 (930.2)	870.0 (988.2)	−545.5 (432.2)	0.252	184.8 (532.9)	0.735

*SD, standard deviation; SE, standard error; 95% CI, 95% confidence interval; HbA1c, glycosylated; SF-36, the 36-item Short Form Survey (SF-36); IPAQ-C, International Physical Activity Questionnaire–Chinese version; MET min week^−1^, minutes of metabolic equivalent per week for physical activity; C-DMQ, the Chinese version of the Diabetes Management Questionnaires*.

a *The statistical description was based on complete cases without imputation*.

b *The within-arm difference was calculated as the difference between 3-month and baseline levels of given measures using the multiply imputed data*.

c *P-value of the within-arm difference was tested by paired t-test and the between-arm difference was tested by Student's t-test*.

d *The between-arm difference was calculated as the difference between intervention and control arms using the multiply imputed data*.

e *The exercise was defined as moderate to vigorous physical activity measured by IPAQ-C regarding weekly frequency and duration*.

Table 4 showed the implementation measures and acceptability scores of the different arms. The intervention arm showed a higher attendance rate (83 vs. 55%) and lower dropout rate (0 vs. 22%), and refusal rate (11 vs. 33%) than the control arm. The correlation of the attendance rate between participants with diabetes and spouses of intervention arm was 0.9 (*P* < 0.01), indicating high concordance. Participants with diabetes of both arms found the interventions acceptable and helpful to improve management knowledge and skills. Correspondingly, spouses of the intervention arm rated the intervention highly (correlated spousal range from −0.2 to 0.7, all *P*s > 0.05), particularly regarding promoted mutual understandings.

**Table 4:** Implementation assessments of the 3-month pilot study (couple pair *N* = 18).

**Variables**	**Participants with diabetes**			**Spouses of intervention arm**		
	**Intervention arm (*n* = 9)**	**Control arm (*n* = 9)**	***P* for arm difference^**a**^**	**Intervention arm (*n* = 9)**	**Spousal correlation coefficient^**b**^**	**P for spousal correlation^**b**^**
**Implementation measures**						
Group education attendance rate (%), mean (SD)	0.8 (0.3)	0.6 (0.4)	0.082	0.8 (0.3)	0.9	0.003
**Booster call follow-ups**						
Dropout rate, *n* (%)	0 (0)	2 (22)	0.470	–	–	
Refusal rate, *n* (%)	1 (11)	3 (33)	0.576	–	–	
**Acceptability scores**^**c**^**, median (25%,75% quartiles)**						
**Taking this course**						
Improved my awareness of diabetes	4.0 (2.5,4.0)	3.0 (3.0,4.0)	0.861	3.0 (3.0,4.0)	0.7	0.065
Improved my control/assistance of blood glucose monitoring	3.0 (3.0,4.0)	4.0 (3.0,4.0)	0.928	4.0 (3.5,4.0)	0.5	0.203
Improved my diabetes management skills	4.0 (3.0,4.0)	3.0 (3.0,4.0)	0.470	4.0 (3.0,4.0)	−0.2	0.721
Increased my awareness of feet care	4.0 (3.0,4.0)	3.0 (3.0,4.0)	0.723	4.0 (4.0,4.0)	0.3	0.453
**Participated in the intervention together with my spouse**						
Helpful for us	4.0 (3.5,4.0)	NA	NA	4.0 (4.0,4.0)	0.7	0.117
Enhanced awareness of diabetes	4.0 (4.0,4.0)	NA	NA	4.0 (3.0,4.0)	0.6	0.090

*SD, standard deviation; NA, not applicable*.

a *P-value of arm difference was tested between participants with diabetes of intervention and control arms by Student's t-test, Fisher test, and Wilcoxon rank-sum test*.

b *Correlation between participants with diabetes and spouses of intervention arm was computed by Pearson correlation and Spearman's rank correlation*.

c *Acceptability was evaluated as the extent to which participants found CCMM were helpful to improve their knowledge and ability to conduct (or to assist, for spouse) diabetes management activities and to promote positive interactions with their partner (for the intervention arm only) on a scale from 1 (strongly disagree) to 4 (strongly agree)*.

Group interviews guided by NPT further provided a structured analysis approach to identify facilitating and inhibiting factors of implementation of CCMM. In terms of coherence and cognitive participation, older couples and health workers all understood the purpose of CCMM and agreed to invest time and energy to practice this model, given its self-reported benefits in improving spousal interactions in daily diabetes management. Community health workers commented that a harmonious marital relationship would be critical to the implementation of CCMM. As for collective action, participants indicated that although the duration and intensity of interventions were generally acceptable, the content of some sessions was overwhelming. Healthcare workers expressed that standardized training to implement CCMM was necessary. In line with acceptability assessments, participants reflected CCMM positively. Health workers additionally suggested that the utilization of online communication tools would facilitate intervention implementation and follow-up.

## Discussion

Our study developed CCMM of T2DM based on theoretical models and tailored to the care needs of older couples with diabetes in China. The constructed model was well-received by health practitioners and public health experts and demonstrated its feasibility in the small-scale pilot, with promising treatment effects on older couples to be further confirmed.

The CCMM was systematically constructed in line with the Medical Research Council's guidance for complex interventions' development and evaluation (27), including theory and evidence identification, needs assessment, and piloting. Previous studies have documented the development process of couple-based interventions on coronary artery disease (28), osteoarthritis (29), low-density lipoprotein cholesterol (30), and heart failure (31). Compared to prior couple-based models (Supplementary Table 4), CCMM was theoretically grounded, with four intervention modules designed correspondingly. While few studies assessed participants with disease and health practitioners' management barriers (30, 32), our intervention contents were tailored to older couples' specific needs and learning capacity. To ensure active spousal participation, CCMM engaged the spouses of participants with diabetes *via* couple-level discussions and skill practices and communal behavior change goals set, which were selectively employed by prior models (15, 28, 30) and innovatively incorporated couple-based behavior change incentives to promote collaboration between couples.

Our pilot generated interesting findings on the treatment effects of CCMM that were worth further validation. Despite statistically non-significant treatment effects identified due to the small sample size, the results also fitted the theoretical model well. In line with the DMCCI, we found that participants with diabetes and spouses in the intervention arm had a higher attendance rate and lower lost to follow-up rate than those in the control arm, which showed that spouses had the positive effect of dyadic coping in helping daily diabetes management (18). Positive dyadic coping brought improvement to self-efficacy of participants with diabetes and spousal efficacy to assist their partners with diabetes, which was shown by the better C-DMQ scores change in the intervention arm than that in the control arm (20). Furthermore, according to the self-efficacy in SCT, better self-efficacy of couples can improve the couple's health and quality of life, which was also shown in the more improvement of several physiological measures and exercise levels among participants with diabetes of the intervention arm than their controlled counterparts, alongside positive changes in spousal mental well-being and exercise levels (19). Previous studies mainly identified the psychological benefits of couple-based interventions, with mixed findings on clinical (13, 14, 30) and behavioral outcomes (14, 15) of people with diabetes, and less is clear about spousal health (16). Our follow-up studies will validate CCMM in a multicenter RCT (17), adopting adequate outcome measures for both the participants with diabetes and their spouses to provide high-quality evidence to the couple-based chronic disease management literature (11).

The current study further advanced the literature by testing the implementation process and analyzing implementation barriers and facilitators. In concordance with a systematic review of trials involving people with a disease and a support person (33), we found higher attendance and retention rates of couple-based than individual-based arms, which may be largely attributed to the spousal companion as indicated by the high spousal concordance score. Trief and colleagues studied challenges in the implementation of couple-based interventions for T2DM management *via* telephone calls. Similar to their study (32), our pilot study revealed challenges of independent assessments and spousal engagement from both intervention recipients' and providers' perspectives and the importance of adequate staff training and monitoring in maintaining intervention fidelity. Our planned RCT will modify implementation strategies incorporating challenges identified: (1) equivalent attention will be paid to recruit, assess, and engage the spouse; (2) care managers will receive 10-h training prior to group education and be equipped with a standardized intervention material package; and (3) education sessions will be recorded by structured fieldnotes and taped for fidelity evaluation.

We constructed CCMM according to the recommended practices and evaluated feasibility in a pilot study with high recruitment and retention rates. However, limited to the small sample size and short duration, the pilot study was underpowered to verify the model's treatment effects. We also did not pay equal attention to collect spouses' blood samples, which would provide objective measures to indicate spousal behavior changes in diet and physical activity. The failure to record spouses' retention rates of booster calls further prevented us from exploring spousal correlations in treatment adherence over time. We set our recruitment age of the couples with diabetes at 60+ in line with China's official retirement age to promote their participation. This approach, however, may lead our study population to an older-aged group that was more likely to be widowed and weaken the model's prevention values for these middle-aged adults at high risks for developing T2DM.

## CONCLUSION

We developed a theoretically grounded couple-based collaborative model for diabetes management, addressing the main management barriers of community-dwelling older Chinese. Large-scale studies with sufficient statistical powers and longer follow-ups are needed to verify the model's long-term effectiveness in improving management behaviors and clinical outcomes of older adults with diabetes and their spouse. This model has the potential of not only enhancing diabetes management activities of older adults with diabetes but also serving as a primary prevention tool for their spouse.

## Data Availability Statement

The raw data supporting the conclusions of this article will be made available by the authors, without undue reservation.

## Ethics Statement

The studies involving human participants were reviewed and approved by Institutional Review Board of the School of Public Health of Sun Yat-sen University. The patients/participants provided their written informed consent to participate in this study.

## Author Contributions

JL, DX, and YL conceptualized the study, analyzed the results, and wrote the first draft of the manuscript. XX, CP, and WY were responsible for designing education and training sessions. TZ oversaw the booster calls. YW conducted the interviews and analyzed NPT result. LO, XC, and XW contributed to the study design and implementation. All authors contributed to revising the manuscript, have read, and approved the final manuscript.

## Conflict of Interest

The authors declare that the research was conducted in the absence of any commercial or financial relationships that could be construed as a potential conflict of interest.

## Footnotes

^1^Wang C, Wu M, Yang Y, Liao J. Effect and quality of couple-based interventions of middle-aged and older adults with type 2 diabetes mellitus: a scoping review (Unpublished) (2020).

## Abbreviations

CCMM: couple-based collaborative management model
RCT: random controlled trials
T2DM: type 2 diabetes mellitus
DMCCI: the dyadic model of coping with chronic illness
SCT: social cognitive theory
HbA1c: glycosylated hemoglobin
BMI: body mass index
NPT: normalization process theory.
