# Supplementary material for: Development and Implementation of Couple-Based Collaborative Management Model of Type 2 Diabetes Mellitus for Community-Dwelling Chinese Older Adults: A Pilot Randomized Trial
Source: Front Public Health. 2021 Jul 13;9:686282. doi: 10.3389/fpubh.2021.686282 (PMC8313732; doi:10.3389/fpubh.2021.686282)
Supplement: Supplementary file 2 [file Table_2.DOCX]

Supplementary Material

**Additional Table 2** The Interview Outlines Following NPT-questions.

| **Components** | **Questions for health practitioners** | **Questions for participants with diabetes** |
| --- | --- | --- |
| **Coherence**  (i.e. sense-making by participants) | - What is your understanding of the couples' collaborative management of diabetes? - What do you think is the difference between the self-management of diabetes and couples' collaborative management of diabetes? - What do you think of the couples' collaborative management of diabetes? What are the benefits? - Will it match the overall goals and activity of the community health service? | - What is your understanding of the management of diabetes with your partner? - What do you think is the difference between the management of diabetes with your partner and by yourself? - What do you think of the management of diabetes with your partner? What are the benefits? - Will it match the overall goals and activity of the community health service? |
| **Cognitive Participation**  (i.e. commitment and engagement by participants) | - Do you think the couples' collaborative management of diabetes is a good idea? - What do you think is the point of the intervention? - Are you going to invest time, energy and work in it? | - Do you think it is a good activity to manage diabetes with your partner? - If you are going to manage diabetes with your partner, what do you think is the key to success? - Are you going to invest time, energy and work in it with your partner? |
| **Collective Action**  (i.e. the work participants do to make the intervention function) | - Will it promote or impede your work? - What effect does this activity have on your life? Can it be adopted in your daily life? - Do you think the 4-week education course is acceptable? - Did you use the materials given and are they worthwhile? How has the use of the materials impacted on the course interaction? - Do you think the time, content, interaction and assignments of the course are appropriate? - Do you think you need extensive training before you can carry out the intervention? - How do the people around you perceive your role in this work? - How do the community hospitals view this project? Will they support your work? - What impact did it have on division of labour, resources, power, and responsibility between community groups and you? | - What effect does this activity have on your life? Can it be adopted in your daily life? - Do you think the 4-week education course is acceptable? - Do you think the time, content, interaction and assignments of the course are appropriate? - Do you think you have the confidence and ability to finish the course and class tasks? - How do the people around you perceive this intervention? Will they support your participation? |
| **Reflexive Monitoring**  (i.e. participants reflect on or appraise the intervention) | - When you have dissatisfaction or questions about the activity, how will you put forward the feedback? - What do you think are ways to improve whole intervention? | - When you have dissatisfaction or questions about the activity, how will you put forward the feedback? - What do you think are ways to improve the whole intervention? |
