# Supplementary material for: Development and Implementation of Couple-Based Collaborative Management Model of Type 2 Diabetes Mellitus for Community-Dwelling Chinese Older Adults: A Pilot Randomized Trial
Source: Front Public Health. 2021 Jul 13;9:686282. doi: 10.3389/fpubh.2021.686282 (PMC8313732; doi:10.3389/fpubh.2021.686282)
Supplement: Supplementary file 3 [file Table_3.docx]

**Appendix 3 Missing Value:**

For physiological health, 3 patients (17%) missed HbA_1c_ in baseline when 2 (11%) people lost in BMI, 3(17%) people lost in lipid profiles, 4 (22%) people lost fasting blood glucose.

For patients’ psychological health, there are 3(17%) patients missing in IPAQ and 1(6%) missing in SF-36 in baseline, and 2 (11%) missing in IPAQ in 3-month follow up. For spouse, 3(17%) missing in IPAQ and DMQ, 2(11%) in SF-36 in baseline when 11% in IPAQ and 6% miss SF-36 in 3-month follow up.

Table 1 Missing value for psychological variables and physiological variables of baseline and 3-month in couples

|  | Participants with Diabetes (N=18) | | Spouse (N=18) | |
| --- | --- | --- | --- | --- |
|  | Baseline | 3-Month | Baseline | 3-Month |
| Psychological variables |  |  |  |  |
| IPAQ, n (%) | 3 (17) | 2 (11) | 3 (17) | 2 (11) |
| SADAC, n (%) | 0 | 0 |  |  |
| C-DMQ, n (%) | 0 | 0 | 3 (17) | 1 (6) |
| SF-36, n (%) | 1 (6) | 0 | 2 (11) | 1 (6) |
| BMI, n (%) | 0 | 2 (11) |  |  |
| Physiological variables |  |  |  |  |
| Fast glauca, n (%) | 0 | 4 (22) |  |  |
| HbA_1c_, n (%) | 3 (17) | 0 |  |  |
| THCO, n (%) | 0 | 3 (17) |  |  |
| TRIG, n (%) | 0 | 3 (17) |  |  |
| LDLC, n (%) | 0 | 3 (17) |  |  |
| HDLC, n (%) | 0 | 3 (17) |  |  |

Abbreviations: HbA1c, glycosylated; FBG, fasting blood-glucose; BMI, [body mass index](file:///D:\enwiki\Body_mass_index); LDL-C, low-density lipoprotein cholesterol; HDL-C, high-density lipoprotein cholesterol; SF-36, the 36-item Short Form Survey (SF-36); SADCA, the Summary of Diabetes Self-Care Activities questionnaire; IPAQ-C, International Physical Activity Questionnaire–Chinese version; C-DMQ, the Chinese version of the Diabetes Management Questionnaires.

As shown in Table 2 and table 3, results of pre-protocol analysis with complete data were robust.

Table 2 Comparison the result of pre-protocol analysis with the result of intention-to-treat analysis in participants and with diabetes (Couple pair N = 18).

| Variables of participants with diabetes | Intervention arm (n = 9) | | Control arm (n = 9) | | | Treatment effect | | |
| --- | --- | --- | --- | --- | --- | --- | --- | --- |
|  | *Difference within arm^3^* | | *Difference within arm^3^* | | | *Difference between arm^4^* | | |
|  | *PP^1^* | *ITT^3^* | *PP^1^* | *ITT^3^* | *PP^1^* | | *ITT^3^* |  |
| Physiological outcomes |  |  |  |  |  | |  |  |
| HbA1c (%) | -0.4 (0.4) | -0.3 (0.5) | -0.1 (0.2) | -0.2 (0.4) | -0.3 (0.4) | | -0.1 (0.6) |  |
| FBG (mmol/L) | 0.0 (1.1) | -0.3 (1.0) | 0.3 (1.0) | 0.3 (0.9) | -0.3 (1.5) | | -0.6 (1.4) |  |
| BMI (kg/m2) | 0.7 (0.4) | 1.0 (0.7) | 0.1 (0.5) | 0.1 (0.5) | 0.6 (0.7) | | 1.0 (0.9) |  |
| Total cholesterol (mmol/L) | -5.4 (5.6) | -4.8 (4.9) | -0.2 (0.3) | -0.2 (0.3) | -5.1 (6.0) | | -4.6 (5.0) |  |
| Triglycerides (mmol/L) | 0.3 (0.4) | 0.2 (0.4) | 0.2 (0.2) | -0.0 (4.7) | 0.2 (0.5) | | 0.2 (0.6) |  |
| LDL-C (mmol/L) | -1.8 (0.3) *** | -1.7 (0.3) *** | -1.1 (0.3) *** | -1.2 (0.3) *** | -0.7 (0.4) | | -0.5 (0.4) |  |
| HDL-C (mmol/L) | 1.7 (0.3) *** | 1.7 (0.3) *** | 0.6 (0.2) *** | 0.7 (0.3) | 1.1 (0.4) *** | | 0.9 (0.4) ** |  |
| Psychosocial outcomes | -0.06 (0.19) |  |  |  |  | |  |  |
| SF-36: Physical component score | 2.1 (2.2) | 0.5 (2.9) | 1 (2.0) | 1.0 (2.0) | 1.1 (2.9) | | -0.5 (3.5) |  |
| SF-36: Mental component score | 0.8 (2.5) | -0.1 (2.5) | -0.6 (0.9) | -0.6 (0.9) | 1.4 (2.5) | | 0.4 (2.6) |  |
| C-DMQ score | 9.7 (5.7) | 9.7 (5.7) | 9.4 (4.8) | 9.4 (4.8) | 0.2 (7.4) | | 0.2 (7.4) |  |
| Behaviour outcomes |  |  |  |  |  | |  |  |
| SADCA score | 4.8 (3.8) | 4.8 (3.8) | 5.8 (5.3) | 5.8 (5.3) | -1.0 (6.5) | | -1.0 (6.5) |  |
| Metabolic equivalent scores measured by IPAQ-C (MET•min•wk-1) | | | | | | | |  |
| Exercise5 | 740 (799.2) | 676.4 (723.8) | -266.7 (688.7) | -62.6 (628.9) | 1006.7 (1102.4) | | 739.0 (961.6) |  |
| Walking | -136.1 (310.0) | -167.6 (294.7) | 506.8 (455.3) | 340.4 (486.9) | -642.9 (538.8) | | -507.9 (572.8) |  |
| Sitting | -240.0 (517.3) | -51.3 (517.7) | 450.0 (413.5) | 378.0 (397.0) | -690.0 (662.3) | | -429.3 (653.6) |  |

Abbreviations: HbA1c, glycosylated; FBG, fasting blood-glucose; BMI, [body mass index](file:///D:\enwiki\Body_mass_index); LDL-C, low-density lipoprotein cholesterol; HDL-C, high-density lipoprotein cholesterol; SF-36, the 36-item Short Form Survey (SF-36); SADCA, the Summary of Diabetes Self-Care Activities questionnaire; IPAQ-C, International Physical Activity Questionnaire–Chinese version; MET•min•wk^-1^, minutes of metabolic equivalent per week for physical activity; C-DMQ, the Chinese version of the Diabetes Management Questionnaires.****:P<0.001,** P <0.01*

Note:

^1^ *PP*: Pre-protocol analysis. The statistical analysis was based on complete data without imputation.

^2^ *ITT*: The Intention-To-Treat. The statistical description was based on all data with imputation.

^3^ Difference within arm means the difference between 3-month and baseline levels of given measures

^4^ Between-arm difference was calculated as the difference between intervention and control arms

Table 3 Comparison the result of pre-protocol analysis with the result of intention-to-treat analysis in participants and with diabetes in spouses (Couple pair N = 18).

| Variables of spouses | Intervention arm (n = 9) | | Control arm (n = 9) | | Treatment effect | |
| --- | --- | --- | --- | --- | --- | --- |
|  | *Difference within arm^3^* | | *Difference within arm^3^* | | *Difference between arm^4^* | |
|  | *PP^1^* | *ITT^3^* | *PP^1^* | *ITT^3^* | *PP^1^* | *ITT^3^* |
| Psychosocial outcomes | |  |  |  |  |  |
| SF-36: Physical component score | -1 (2.8) | -1.0 (2.9) | 1.8 (2.6) | 0.8 (2.6) | -2.8 (3.8) | -1.8 (4.0) |
| SF-36: Mental component score | -1.8 (1.8) | -2.0 (2.1) | -4.4 (1.9) | -5.1 (2.0) | 2.6 (2.6) | 3.1 (2.8) |
| C-DMQ score | 3.4 (12.2) | 10.0 (12.8) | -10.5 (10) | -11.3 (9.8) | 13.9 (15.6) | 21.3 (16.1) |
| Behaviour outcomes | |  |  |  |  |  |
| Exercise5 | 1060 (724.2) | 1131.2 (607.0) | 745.7 (1051.5) | 695.0 (869.0) | 314.3 (1276.) | 436.3 (1056.1) |
| Walking | -379.5 (638) | -454.8 (564.3) | -1122 (532.1) | -1043.1 (484.3) | 742.5 (830.8) | 588.3 (737.7) |
| Sitting | -210 (254.3) | -360.7 (308.6) | -690 (499.3) | -545.5 (432.2) | 480 (591) | 184.8 (532.9) |

Abbreviations: SF-36, the 36-item Short Form Survey (SF-36); IPAQ-C, International Physical Activity Questionnaire–Chinese version; MET•min•wk^-1^, minutes of metabolic equivalent per week for physical activity; C-DMQ, the Chinese version of the Diabetes Management Questionnaires. ****:P<0.001,** P <0.01*

Note:

^1^ *PP*: Pre-protocol analysis. The statistical analysis was based on complete data without imputation.

^2^ *ITT*: The Intention-To-Treat. The statistical description was based on all data with imputation.

^3^ Difference within arm means the difference between 3-month and baseline levels of given measures

^4^ Between-arm difference was calculated as the difference between intervention and control arms
