# Supplementary material for: Development and Implementation of Couple-Based Collaborative Management Model of Type 2 Diabetes Mellitus for Community-Dwelling Chinese Older Adults: A Pilot Randomized Trial
Source: Front Public Health. 2021 Jul 13;9:686282. doi: 10.3389/fpubh.2021.686282 (PMC8313732; doi:10.3389/fpubh.2021.686282)
Supplement: Supplementary file 4 [file Table_4.docx]

Supplementary Materia

**Additional Table 4** Comparison of intervention development and contents.

| **Interventional model** | **Couple-based Collaborative Management Intervention**  **(CCMM)** | **Self-management Using Couples' Coping Enhancement in Diseases.**  **(SUCCEED) (12)** | **Couples Partnering for Lipid Enhancing Strategies (CouPLES) (25)** | **Couple-oriented education and support (29)** | **Partners for life intervention (30)** |
| --- | --- | --- | --- | --- | --- |
| **Targeted health condition** | Type 2 diabetes mellitus (T2DM). | Heart failure (HF). | Low-density lipoprotein cholesterol. | Hip or knee osteoarthritis. | Coronary artery disease. |
| **Theoretical model** | Berg and Upchurch's Dyadic Model of Coping with Chronic Illness (DMCCI)  Bandura's Social Cognitive Theory (SCT). | Leventhal's Self-regulatory Model of Illness Behaviour  Bandura's Unifying Theory of Self-efficacy. | Not specified. | Not specified. | Baucom's Cognitive Behavioural Couples Therapy (CBCT).  Deci and Ryan's Self- Determination Theory.  Prochaska and DiClemente's Transtheoretical Model of Behavioural Change. |
| **Users' barriers assessments** | Group interviews were conducted among older couples & health workers. | Semi-structured interviews Conducted with couples managing HF and clinical providers. | None. | None. | None. |
| **Incorporation of** **experts' opinion** | Delphi approach with a panel of healthcare specialists. | Consultation with the local Patient Education Coordinator and Director of the Office of Education. | None. | None. | None. |
| **Intervention modules & Implementation characteristics** | Health Education & Training:  a) Diabetes & complications management.  b) Healthy diet.  c) Medication adherence.  d) Exercise.  Behaviour Change Booster Calls.  Behaviour Change Incentive. | Telephone calls:  a) Manage interpersonal relationship & relationship stress.  b) Manage HF and caregiver burden manage negative emotions.  c) The problem-solving pursuit of pleasant activities & strategies to maintain change. | Telephone calls:  a) Medication adherence  b) Diet.  c) Exercise.  d) Patient-physician communication. | Group education:  a) The aetiology and treatment of arthritis.  b) Self-management strategies for managing pain and strengthening joints.  c) The benefits of exercise.  d) Communication skills.  e) Ways to cope with negative emotions effectively.  Booster calls to review couples' progress. | Group education:  a) Nutrition.  b) Exercise.  c) Medication adherence.  d) Maintenance & Relapse prevention. |
| **Implementation contex**t | Community health care centres. | Participants' home. | Veterans Affairs Medical Centre. | Rheumatology clinics. | Hospitals |
| **Delivery methods** | Group education.  Booster telephone calls. | Telephone calls. | Telephone calls. | Group education.  Booster sessions via telephone. | Group education. |
| **Duration & Frequency** | Four-weekly 1.5-hour group education sessions.  2-month weekly booster calls tailored to patients' behaviour change barriers. | Six 45–60 min sessions. | Monthly telephone calls at every month except for months 6 and 11. | Six weekly 2-hr sessions. | 18 sessions (12 weekly sessions followed by six alternative week sessions) over a total of 24weeks. |
| **Intervention personnel** | Community health workers. | Trained facilitators who were research assistants. | Nurse. | Research assistants. | Trained therapists who were engaged in project. |
| **Special Intervention to spouses** | couple-level discussions and skill practices.  Communal behaviour change goals setting.  Couple-based behaviour change incentives. | Received education and resources for caregivers in managing HF.  Emphasized the importance of managing spousal caregivers' well-being and self-care.  Identified a reasonable and desirable behaviour. | Informed of patients' goals.  Provided with strategies to support patients.  Provided with printed educational materials plus the CouPLES intervention. | Set health-related goals for the following week.  The practice of cognitive or behavioural pain management strategies.  Reducing negative emotional contagion between partners.  Provided couples' strategies for managing medications. | Couple-level discussions & Skills practice.  Motivation and communication skills training (couples group only). |

**Reference**

1. Trief PM, Fisher L, Sandberg J, Cibula DA, Dimmock J, Hessler DM, et al. Health and Psychosocial Outcomes of a Telephonic Couples Behavior Change Intervention in Patients With Poorly Controlled Type 2 Diabetes: A Randomized Clinical Trial. Diabetes Care. 2016;39(12):2165-73. doi:10.2337/dc16-0035

2. Voils CI, Yancy WS, Jr., Kovac S, Coffman CJ, Weinberger M, Oddone EZ, et al. Study protocol: Couples Partnering for Lipid Enhancing Strategies (CouPLES) - a randomized, controlled trial. Trials. 2009;10:10. doi:10.1186/1745-6215-10-10

3. Martire LM, Schulz R, Keefe FJ, Rudy TE, Starz TW. Couple-Oriented Education and Support Intervention for Osteoarthritis: Effects on Spouses' Support and Responses to Patient Pain. Fam Syst Health. 2008;26(2):185-95. doi:10.1037/1091-7527.26.2.185

4. Sher TG, Bellg AJ, Braun L, Domas A, Rosenson R, Canar WJ. Partners for Life: a theoretical approach to developing an intervention for cardiac risk reduction. Health Educ Res. 2002;17(5):597-605. doi:10.1093/her/17.5.597
